# Supplementary material for: The interactome of intact mitochondria by cross-linking mass spectrometry provides evidence for coexisting respiratory supercomplexes
Source: Mol Cell Proteomics. 2017 Dec 8;17(2):216–32. doi: 10.1074/mcp.RA117.000470 (PMC5795388; doi:10.1074/mcp.RA117.000470)
Supplement: Supplemental Data [file supp_RA117.000470_133922_0_supp_23978_fzffwf.zip › spectra_annotation/mito_DR_spectra_annotation/42-1-5-1-6-1.pdf]

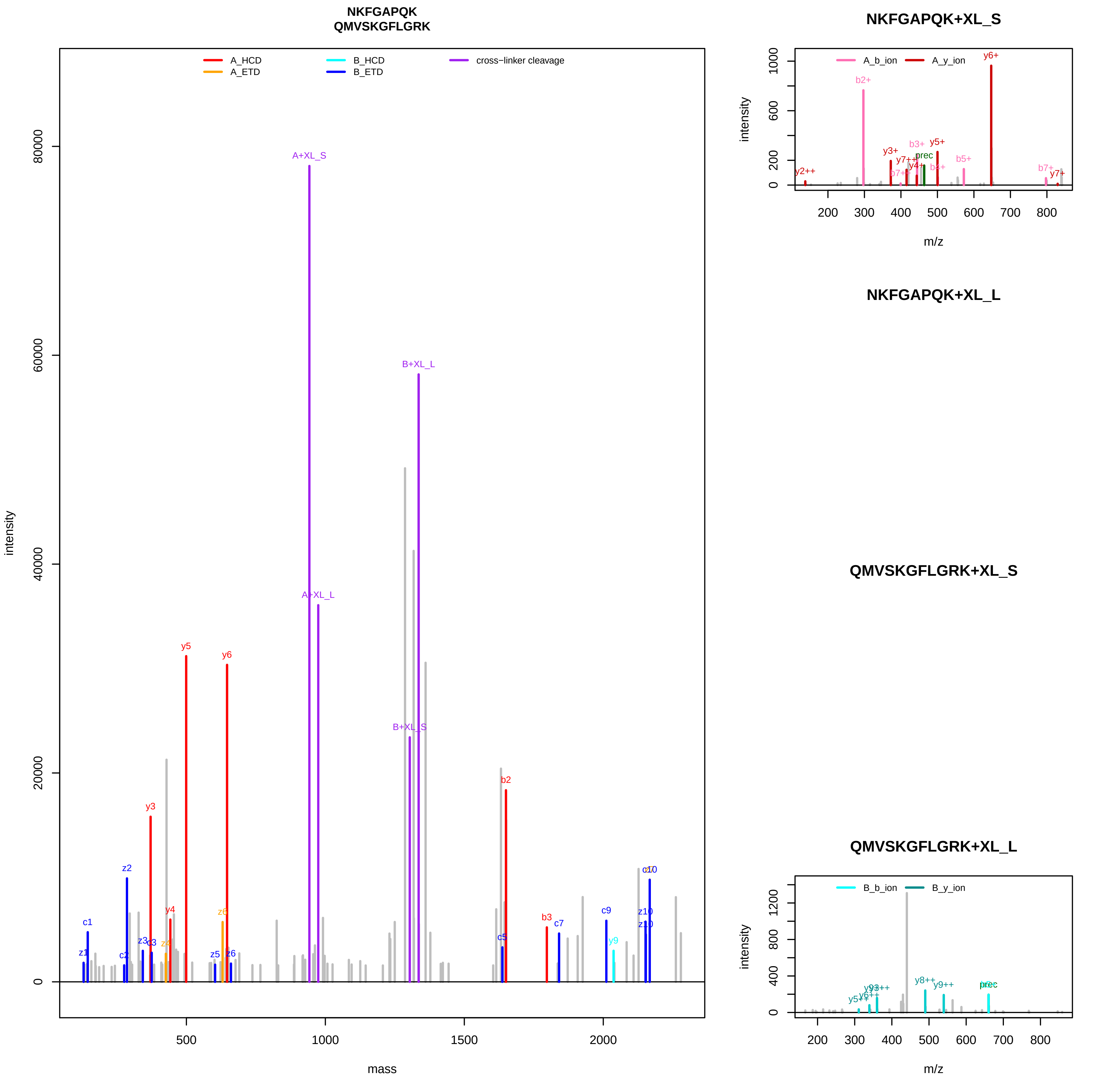

NKFGAPQK
QMVSKGFLGRK
A_HCD
A_ETD
B_HCD
B_ETD
cross−linker cleavage
A+XL_S
B+XL_L
A+XL_L
B+XL_S
y5
y6
y3
y4
b2
b3
z2
c1
z1
c2
z3
c3
z4
z5
z6
c5
c7
c9
y9
z10
c10
intensity
mass
NKFGAPQK+XL_S
A_b_ion
A_y_ion
y6+
b2+
y3+
b3+
y5+
y7++
prec
b5+
y2++
b7+
y7+
m/z
NKFGAPQK+XL_L
QMVSKGFLGRK+XL_S
QMVSKGFLGRK+XL_L
B_b_ion
B_y_ion
y8++
y9++
prec
y5++
y6++
